# Uncommon Female-Predominant Etiologies of Cryptogenic Stroke

**DOI:** 10.3389/fneur.2022.900991

**Published:** 2022-06-24

**Authors:** Jing Dong, Xin Ma

**Affiliations:** ^1^Department of Neurology, Xuanwu Hospital, Capital Medical University, Beijing, China; ^2^National Clinical Research Center for Geriatric Disorders, Beijing, China; ^3^Clinical Center for Cardio-Cerebrovascular Disease of Capital Medical University, Beijing, China

**Keywords:** female, etiology, uncommon, stroke, cryptogenic

## Abstract

The etiologies of cryptogenic stroke are complex and heterogeneous. A number of uncommon etiologies are not fully recognized, some of which predominantly affect females. Most of these etiologies are closely related to the hormonal level, reproductive factors, coagulation function, and medications of females. Moreover, once cryptogenic stroke is diagnosed, females tend to have worse outcomes. Therefore, prompt etiological recognition and treatment are crucial for good recovery. The aim of this article is to review advances in exploring uncommon female-predominant etiologies of cryptogenic stroke. These etiologies are categorized into arterial, cardiac, and venous sources. Arterial vasoconstrictive narrowing, intimal injury, and intimal developmental abnormality can cause brain ischemia or artery-to-artery cerebral embolism. Myocardial contraction dysfunction, cardiac wall injury, and developmental abnormality can induce intracardiac thrombosis and lead to cardiac embolism. In addition, cortical venous thrombosis and occult venous thromboembolism *via* intracardiac or extracardiac channels also account for cryptogenic stroke in females. Due to the lack of knowledge, in clinical practice, the above etiologies are seldom assessed. The low incidence rate of these etiologies can lead to missed diagnosis. This review will provide novel clinical clues for the etiological diagnosis of cryptogenic stroke and will help to improve the management and secondary prevention of stroke in the female population. In the future, more studies are needed to explore the etiology and prevention strategies of cryptogenic stroke.

## Introduction

The etiologies of cryptogenic stroke are complex and heterogeneous. A number of uncommon etiologies are not fully recognized, some of which predominantly affect females. Studies have shown that differences in hormonal factors, coagulation state, and immunity may predispose females to stroke at certain physiological periods, including pregnancy and the peripartum period (1, 2). Once cryptogenic stroke is diagnosed, females tend to have worse outcomes (1, 3), therefore prompt etiological recognition and treatment are crucial for good recovery. However, these potential etiologies are often undiagnosed in clinical practice due to the limitation of knowledge and examination technologies. In this article, we will review the advancement in exploring uncommon female-predominant etiologies of cryptogenic stroke, which will provide novel clinical clues for the etiological diagnosis of cryptogenic stroke and might be helpful in improving the diagnosis and secondary prevention of stroke as a whole.

## Arterial Sources of Cerebral Infarction or Embolism

### Vascular Sources of Cerebral Infarction or Embolism

Atherosclerotic arterial stenosis/occlusion and vulnerable plaque eruption are predominant pathogenic mechanisms of ischemic stroke (23). However, some uncommon conditions, including cerebral vasoconstrictive narrowing and thromboembolism caused by arterial intimal injury or intimal developmental abnormality, are unrecognized. These conditions are closely related to the hormonal level, gestational status, and medications of females, which deserve more attention from clinicians.

#### Reversible Cerebral Vasoconstriction Syndrome

Reversible cerebral vasoconstriction syndrome (RCVS) refers to a group of disorders characterized by reversible vasoconstriction of the cerebral vasculature, including uncommon conditions like drug-induced angiopathy and postpartum angiopathy… RCVS. should be highly suspected in patients with recurrent thunderclap headache, multifocal segmental cerebral artery narrowing typically showing a beaded appearance on angiography, and after exclusion of diseases with similar clinical manifestations including arterial dissection, aneurysm, and vasculitis. In addition, the arterial narrowing in RCVS is characteristically reversible within 3 months (4).

In previous studies, up to 64.2–85.6% of RCVS patients were female (4, 5), while only 14.4–35.8% were men. The predominance of females in RCVS might be associated with the use of vasoactive medications including triptans, selective serotonin reuptake inhibitors, or noradrenergic and selective serotonergic antidepressants, or with postpartum and eclampsia (6, 7). Vasoactive substances and surge of blood pressure can induce sympathetic overactivation and subsequently persistent vasoconstriction. Estrogen also participated in the deregulation of cerebral vascular tone (4). Further, placental growth factor, soluble fms-like tyrosine kinase-1, and transforming growth factor β might be implicated in developing postpartum RCVS (2, 4), the mechanism of which is still unclear.

It is usually thought that RCVS has a benign course, but in fact, around 6–39% of RCVS cases developed ischemic stroke (4). Particular attention should be paid to RCVS-related stroke in pregnancy and the postpartum periods. Recently, two retrospective studies have indicated that RCVS might be the most common cause of stroke during these periods. Moreover, the condition of RCVS-related stroke in these periods might continuously worsen and even lead to death (24–26). It should be noted that the prodromal headache symptom of RCVS-related stroke is sometimes atypical or even absent (27), which would lead to misdiagnosis of RCVS as atherosclerotic stenosis or aneurysm, or missed diagnosis. Thus, for females with cryptogenic stroke, particularly for those in pregnancy and the postpartum periods, not only should detailed history taking of prodromal headache symptom, precipitating factors, and medications be taken, but also dynamic observation of the reversible changes of cerebral vasculature should be highlighted, in order to clarify the diagnosis.

#### Pregnancy Associated Aortic Dissection

Aortic dissection is a known uncommon etiology of cryptogenic stroke, but surprisingly, for young and middle-aged female, over half of aortic dissections developed in pregnancy (8). Particularly, pregnant females with multiple gestation, connective tissue disorders, gestational diabetes, gestational hypertension and pre-eclampsia/eclampsia are more prone to pregnancy-associated aortic dissection (PAD) (28, 29). PAD might develop under the synergistic effect of various factors. The increase in heart rate, cardiac output, and abdominal pressure in pregnancy would add forces to the aortic wall. In addition, changes in estrogen level during pregnancy might be associated with the weakening of elastic fibers and medial degeneration of the aortic wall (28). These factors combined could increase the risk of aortic intimal laceration.

Studies have shown that up to 76% of PAD were Stanford type A, which means the dissection might involve the orifices and proximal segments of the three major branches of aorta and would lead to stroke and upper limb ischemia (30, 31). Moreover, PAD-related stroke is a rapidly progressive life-threatening condition with a high rate of maternal and fetal death, which requires urgent diagnosis and treatment. Patients with PAD might sometimes present with atypical chest pains. Moreover, the characteristic imaging features of dissection, including entrance and exit tears, true and false lumen, and intimal flap, are easily neglected in routine examinations for stroke, such as carotid ultrasonography and echocardiogram, which would delay or mislead the diagnosis of PAD-related stroke. Therefore, for pregnant females with cryptogenic stroke, clinicians should be highly alert to PAD even when encountering atypical manifestations.

#### Intracranial Arterial Dissection

Unlike vertebral and carotid artery dissections, fewer than 20% of stroke-related dissections involve intracranial arteries, including dissections of the basilar artery, posterior inferior cerebellar artery, and arteries around the circle of Willis, which are not fully recognized. The smaller diameters of these arteries limit the ability of routine vascular imaging examinations to detect characteristic imaging features of dissection, including true and false lumen and intimal flap; the dissection tends to be missed or misdiagnosed as aneurysm, vasculitis, or fibromuscular dysplasia, etc. (32, 33).

It is worth noting that among East Asian patients, up to 67–78% of cervicocranial arterial dissections are intracranial arterial dissection (IAD), and around half of IAD patients are female (9). In this regard, when east Asian females with cryptogenic stroke presented with dissection related factors, including prodromal headache and medical history of head trauma or violent movement, while no evidence of carotid or vertebral artery dissection was found, high resolution magnetic resonance imaging or thin slice spiral CT should be applied to detect IAD.

#### Carotid Web

Carotid web (CW) refers to a thin shelf-like projection developed from the posterior wall of the carotid bulb, which was demonstrated to be the fibroelastic thickening of the intima in histopathological examinations (34). Researchers hypothesized that CW might be a rare and atypical subtype of fibromuscular hyperplasia with unknown pathogenesis (10).

In recent years, studies have found a close relationship between CW and the incidence and recurrence of cryptogenic stroke in young and middle-aged adults. CW has been detected in 9.4–37% of young and middle-aged cryptogenic stroke patients; the percentage was markedly higher than that of non-stroke controls (1–7%) (35). In addition, a cohort study of cryptogenic stroke patients has reported that 29% of CW ipsilateral to the infarction developed mural thrombi; during 1-year follow-up, 32% of stroke patients had recurring cerebral infarctions in brain regions ipsilateral to the CW (11). Researchers hypothesized that the pathogenic mechanism of CW-related stroke might be a cerebral embolism due to dislodgement of mural thrombus formed within CW.

Particularly, in recent meta-analyses and case control studies, up to 61–91% of CW-related stroke patients were female (10–13). The underlying mechanism of developing CW in females is still not clear, although genetic, hormonal factors, and vascular injury might play a role. When evaluating for cryptogenic stroke in young and middle-aged females, careful examination of the posterior wall for a protrusion along the carotid bulb should be done on computed tomography angiography or magnetic resonance angiography.

#### Aortic Mural Thrombus

While mural thrombus due to aortic atherosclerotic plaque rupture is a known source of cryptogenic stroke, less attention has been given to aortic mural thrombus (AMT) with no underlying aortic diseases. The pathogenesis of AMT is unclear, although coagulation disorders and malignancy have both been reported. The pathogenesis of this AMT is still unclear; some cases also have coagulation disorders, hematologic disorders, malignancy, inflammatory bowel disease, or are under chemotherapy or taking steroids or oral contraceptives (14), which suggests a possible link with hypercoagulable state. Intimal injuries secondary to smoking and trauma might also induce aortic thrombosis (36). Although only around 200 AMT cases were reported to date (14), over half of the patients were female, and about 88% of AMT were movable floating thrombus with potentially high risk of embolism. Moreover, about 4–14% of the embolic events were stroke (14, 37). Therefore, if the aortic origin of cryptogenic cerebral embolism was suspected, especially in females with hypercoagulation disorders and systemic embolism, the aorta should be examined for AMT by transesophageal echocardiogram or contrast enhanced computed tomography even when atherosclerosis was not evident.

### Cardiac Thromboembolism

Cardiac thrombi secondary to atrial fibrillation, recent myocardial infarction, systolic heart failure, and prosthetic heart valves are common sources of cerebral embolism. In addition, infective endocarditis, papillary fibroelastoma, and myxoma are relatively uncommon but known sources of cardioembolism (15). Apart from these, some uncommon diseases compromising the structure and function of the heart can cause cardiac thrombi s as well. As most symptoms of these diseases are non-specific, they tend to be misdiagnosed or neglected, particularly when accompanied by common heart diseases. Moreover, this group of diseases are difficult to detect *via* routine transthoracic echocardiogram; transesophageal echocardiogram or other high-resolution imaging techniques are usually needed to clarify the diagnosis. In these situations, the etiology of stroke would be underdiagnosed. Several of these diseases are female-predominant, which call for special attention.

#### Takotsubo Syndrome

Takotsubo syndrome is an acute cardiac syndrome characterized by transient and reversible left ventricular contraction dysfunction affecting more than one coronary artery territory. The incidence of TTS was reported to be approximately 10/100,000. TTS patients frequently suffered from emotional or physical stress prior to symptom onset and have clinical and electrocardiographic manifestations similar to that of acute coronary syndrome; in a few cases, patients might present with a chronic course or remain asymptomatic. On left ventriculography, TTS characteristically shows an apical ballooning appearance due to dyskinetic apical contraction and compensatively enhanced basal wall contraction of the left ventricle. The ventricle configuration and function usually recovered spontaneously within days or months (16).

TTS is an uncommon etiology of stroke and could be the underlying etiology of cryptogenic stroke. According to previous studies, the incidence of stroke among TTS patients admitted to the hospital, 30 days and 1 year after disease onset, were 1–1.7, 2.8, and 4.2%, respectively (17). The pathogenic mechanism of TTS-related stroke might be cardioembolism secondary to ventricular thrombosis, as left ventricular thrombi were detected in about 1.3–5.3% of TTS cases (38). Once the ventricle function returned to normal, the thrombi might dislodge from the ventricle wall after myocardial contraction and cause cerebral embolism. Whereas, since the clinical manifestations of TTS mimics that of acute coronary syndrome, and sometimes even no obvious symptoms are presented, TTS tends to be misdiagnosed or missed in clinical practice.

Up to 70–90% of TTS patients and most of the recurring TTS cases were female (16, 18, 39); in addition, 10% of hospitalized female patients who initially presented with suspected acute coronary syndrome were ultimately diagnosed with TTS (16). It should be noted that most of the females with TTS were postmenopausal, which means TTS might be explained by the hormonal changes after menopause. Catecholamine cardiotoxicity plays a key role in the pathogenesis of TTS. Emotional and physical stressors can induce sympathetic hyperactivation and surge of circulating catecholamines, which would cause myocardial stunning and coronary spasm, and ultimately lead to ventricular contraction dysfunction. It is well-established that estrogen protects against the cardiotoxicity of catecholamine; as the estrogen level decreases after menopause, its protective effect attenuates, which would render the heart vulnerable to TTS in response to stress (16).

When diagnosing cryptogenic stroke in females, especially in postmenopausal females, detailed inquiry of predisposing stressful events would be helpful. If prodromal acute chest pain is presented, careful discrimination of TTS from acute coronary syndrome would be necessary.

#### Left Atrial Appendage Aneurysm

Left atrial appendage aneurysm (LAAA) refers to the abnormal aneurysmal enlargement of the left atrial appendage. The pathogenesis of LAAA is undetermined; 90% of LAAA cases were congenital, probably due to congenital dysplasia of the atrial pectinate muscles, others were secondary to mitral valve diseases, or other conditions leading to elevated left atrial pressure. Most LAAA patients were asymptomatic, while others presented with palpitation, dyspnea, or chest pain. In histopathological examinations, endocardium or myocardium fibrosis of left atrial appendage was demonstrated. The enlarged aneurysm might compress the left coronary artery and result in myocardial ischemia and atrial arrhythmia including atrial fibrillation/flutter (26.7%) and supraventricular tachycardia (9.9%) (40). Of the LAAA cases, 5.9% developed thromboembolic events (40), and mural thrombi were detected attaching to the wall of left atrial appendage in published LAAA-related stroke cases (19, 41). LAAA is rare, only around 100 cases were reported in literature, although 52.5% of patients were female. Thus, for females with cryptogenic stroke, particularly for those presenting with palpitation, dyspnea, or arrhythmia, echocardiogram should be performed to assess for LAAA.

#### Left Atrial Dissection

Left atrial dissection (LAD) refers to the blood-filling false cavity extending from the mitral annular area to the left atrium free wall or interatrial septum. Most LAD cases were reported after cardiac surgery, such as mitral valve replacement, radiofrequency ablation, coronary bypass surgery, or percutaneous coronary angioplasty, while no more 1/8 of cases were spontaneous. LAD tended to occur in the posterior atrial wall; the false cavity might gradually enlarge and grow into a hematoma, which would obstruct the mitral valve inflow or orifices of pulmonary veins and induce thrombosis. The clinical manifestations of LAD vary widely, including dyspnea, hemodynamically instable symptoms, chest pain, arrhythmia, dysphagia, and stroke (20, 42). In most cases, the symptom presented within hours or days post-surgery (73.3%), while a few presented after months or years. About half of LAD patients were hemodynamically instable and presented with congestive heart failure and low-output syndrome, while 15% were asymptomatic (42). Although no more than 100 LAD cases were reported, over half of patients were female. In this regard, LAD should be counted as another rare etiology of cardioembolism in females. Particularly for female patients who had a recent history of cardiac surgery prior to stroke onset and presented with hemodynamically unstable symptoms, LAD should be considered. The imaging features of LAD are not easily distinguishable from that of cardiac tumor or pericardial effusion in routine transthoracic echocardiogram and magnetic resonance imaging, when suspecting LAD, transesophageal echocardiogram is possibly needed to clarify the diagnosis (20).

## Venous Sources of Cerebral Infarction and Embolism

Venous thrombi cause stroke in two ways, either by direct occlusion of cerebral drainage veins or by indirect paroxysmal thromboembolism *via* intracardiac or extracardiac channels. Apart from cerebral venous sinuses and lower limb veins, some venous thrombi form in uncommon locations, which are almost undetectable by routine vascular examinations and are closely associated with a hypercoagulable state. Since females are prone to hypercoagulation disorders, it is necessary to screen for occult venous thrombi for females with cryptogenic stroke.

### Isolated Cortical Venous Thrombosis

It is known that infarction due to cerebral venous sinus thrombosis is an uncommon type of stroke. Unexpectedly, stroke due to isolated cortical venous thrombosis (ICVT) is even more uncommon and unrecognized. In previous reports, only 6% of intracranial venous thrombosis were ICVT (43, 44). The clinical manifestations of ICVT were similar to that of cerebral venous sinus thrombosis, but intracranial hypertension was seldom presented (44). The diagnosis of ICVT-related stroke is often difficult, as the imaging features of ICVT are not evident in routine vascular imaging examinations due to the small diameter of involved cortical veins. Magnetic resonance venography, black-blood magnetic resonance imaging, or CT venography is always needed to clarify the diagnosis.

According to the meta-analysis conducted in 2014, up to 2/3 of ICVT patients were females (44). The development of ICVT in females might be associated with a hypercoagulable state. In this study, 21% of female had histories of taking contraceptives and 35% were pregnant or in the postpartum period (44), both conditions were closely related to hypercoagulation. In addition, intracranial hypotension might also be an inducing factor of ICVT in females. In a retrospective study recruiting 51 ICVT patients, 10 patients (23.3%) had intracranial hypotension, while intracranial hypotension was present in merely 2% of all patients with cerebral venous thrombosis. Among the 10 patients, seven patients were female while only three were men (21).

In clinical practice, when encountering a female stroke patient with a history of taking contraceptives, pregnant or in the postpartum period, or with intracranial hypotension, particularly those with isolated cortical lesion, ICVT should be suspected.

### Paradoxical Emboli

#### May-Thurner Syndrome

May-Thurner syndrome (MTS) is characterized by the impairment of iliac venous return and obstruction of the iliac vein due to compression by the overlying iliac artery against the lumbar spine. Although the compression is a common anatomic variation found in 14–32% of cadavers (45), and most MTS patients were asymptomatic or with only insignificant presentations like mild lower limb edema and pigmentation, it still calls for attention. In a meta-analysis of 1,569 symptomatic MTS cases, 52.4% of patients had deep venous thrombosis and 7.8% had pulmonary embolism (46). A hypercoagulable state might be an inducing factor of MTS-related venous thrombosis. In this study, 11.2% of MTS patients had malignant tumor, 16.9% had a history of recent surgery, 4.6% had trauma, and 9.6% had coagulation disorders including factor V Leiden thrombophilia, protein C, and protein S deficiency (46).

MTS tends to develop in females. In previous studies, about 2/3 of symptomatic MTS patients were female, which might be associated with pregnancy, taking contraceptives, or estrogen replacement therapy. Moreover, the condition of females with MTS tended to be more severe than men; it was reported that females had a higher percentage of severe iliac venous stenosis (19.5 vs. 11.1%) (47) and pulmonary embolism (9.9 vs. 1.6%) compared with men (46).

MTS might not only cause pulmonary embolism, but also lead to stroke. One of the recent studies utilizing magnetic resonance imaging to examine the pelvic veins of cryptogenic stroke patients has found that 31% of cryptogenic stroke patients had MTS, which was significantly higher than that of controls (10%). The compression degree of iliac veins in cryptogenic stroke patients with MTS was also higher than that of controls (32 vs. 13%) (22). Another study had retrospectively analyzed 50 cryptogenic stroke cases with patent foramen ovale (PFO) and found that 10% of patients had MTS and 8% had pelvic venous thrombosis, while none of the patients had lower limb venous thrombosis (48). It is known that the patent foramen ovale is the main conduit from which the venous thrombi crosses directly into the systemic circulation and causes cerebral embolism. This study indicated that pelvic venous thrombi secondary to MTS might be the origin of paraxysmal cerebral embolism apart from lower limb venous thrombi, and MTS might be the potential etiology of crytogenic stroke.

Therefore, for female patients with cryptogenic stroke with PFO, especially those with hypercoagulation disorders, pregnant, or taking oral contraceptives, the use of RoPE (Risk of Paradoxical Embolism) score and PASCAL (PFO-Associated Stroke Causal Likelihood) classification system to evaluate the association between stroke and PFO and the screening for MTS by contrast enhanced magnetic resonance imaging or contrast enhanced computed tomography of pelvic venous thrombi would be necessary.

#### Pulmonary Arteriovenous Malformation

Apart from patent foramen ovale, which is the most common etiology of paraxysmal cerebral embolism, in a few conditions, venous thrombi can flow through the vascular bed of pulmonary arteriovenous malformation (PAVM) into systemic circulation. The incidence of PAVM was reported to be 2–3/100,000; female patients outnumber male with a female to male ratio of 1.5–1.8:1, and more than half of PAVM patients had hereditary hemorrhagic telangiectasis (49). While most PAVM patients were asymptomatic, 30% developed hypoxemia, stroke, brain abscess, migraine, decompression illness, hemoptysis, or hemothorax. In previous studies, about 3.2–55% of PAVM patients developed stroke (49, 50). The pathogenic mechanism of stroke might be paraxysmal cerebral embolism caused by venous thrombi passing through the abnormal communication between pulmonary artery and veins and ultimately into the left heart chamber and arterial system.

Special attention should be paid to PAVM in pregnancy. Studies have observed that, in pregnancy, PAVM tended to increase in volume and number (49, 51), which might be related to the elevation of cardiac output in this period (52).

Therefore, for female patients with cryptogenic stroke, when paraxysmal cerebral embolism was suspected but no intracardiac shunting was demonstrated, for instance, when the transcranial doppler bubble test was positive but no patent foramen ovale was found in transesophageal echocardiogram, PAVM should be screened. Particularly for female patients with a family history of hemorrhagic telangiectasis or with recurrent epistaxis or multifocal cutaneous telangiectasis, pre-pregnancy screening for PAVM would be helpful in preventing severe complications like stroke in pregnancy.

## Discussion

Various uncommon stroke etiologies exist in the female population that still remain unrecognized. The incidence rates of these etiologies are generally low, for instance, the incidence of TTS and PAVM are 10/100,000 and 2–3/100,000, respectively, and no more than 200 cases of AMT, LAAA, and LAD were reported in the literature. The development of these etiologies are closely related to the hormonal profiles in different physiological stages of female life, coagulation function, and medications. In pregnancy and postpartum, the changes of estrogen and placental-related hormones might influence the regulation of cerebral vascular tone, and induce aortic wall degeneration, which would predispose females to RCVS and PAD. In addition, the hormonal changes might promote hypercoagulability, and subsequently induce thrombosis in uncommon locations as seen in AMT, ICVT, and MTS. In post menopause, the decrease in estrogen level attenuates its protective effect against the cardiotoxicity of catecholamine, which would render the heart more vulnerable to stress and predispose females to TTS. In clinical practice, for females with cryptogenic stroke, detailed medical history taking, comprehensive coagulation tests, and special imaging applications are vital for detecting occult thrombi, dissection, and vascular/cardiac developmental abnormalities that might be easily missed in routine examinations (the screening recommendations for uncommon female-predominant etiologies of cryptogenic stroke are listed in Table 1). Currently, etiological studies focusing on females with cryptogenic stroke are scarce. In the future, greater efforts should be made to explore the etiology and prevention strategies of cryptogenic stroke in the female population, so as to improve the diagnosis and treatment of stroke.

**Table 1:** Proportion of women vs. men and screening recommendations for uncommon female-predominant etiologies of cryptogenic stroke.

**Etiology**	**Proportion of women vs. men**	**Clinical history clues**	**Diagnostic testing clues**
**Arterial sources**			
**Vascular sources**			
Reversible cerebral vasoconstriction syndrome	64.2–85.6% vs. 14.4–35.8% (4, 5)	1. Prodromal thunderclap headache 2. Medical history of migraine, eclampsia 3. Postpartum or stage of pregnancy 4. Vasoactive medications including triptans, selective serotonin reuptake inhibitors, noradrenergic and selective serotonergic antidepressants, cannabis, binge drinking, etc. (6, 7)	1. Multifocal segmental cerebral artery narrowing with beaded appearance on DSA//MRA/CTA/contrast-enhanced vessel wall imaging 2. Complete or substantial resolution of vasoconstriction within 3 months of clinical onset
Pregnancy associated aortic dissection	100% (60% in pregnancy and peripartum) vs. 0 (8)	1. Chest pain or back pain, hemodynamically instable symptoms, asymmetric brachial arterial pressure 2. Medical history of multiple gestation, connective tissue disorders, gestational diabetes, gestational hypertension and pre-eclampsia/eclampsia 3. Stages of pregnancy or postpartum	1. Entrance and exit tears, true and false lumen, intimal flap on CTA/DSA/TEE
Intracranial arterial dissection	33–42% vs. 58–67% (9)	1. Age 2. Ethnicity 3. Prodromal headache 4. Recent medical history of head trauma, violent movement	1. True and false lumen, intimal flap on high resolution MRI/ thin- slice spiral CT
Carotid web	61–91% vs. 9–39% in carotid web related stroke patients (10–13)	1. Age	1. Thin shelf-like filling defect attached to the wall of carotid bulb on carotid ultrasonography/MRA/CTA
Aortic mural thrombus	53 vs. 47% (14)	1. Medical history of coagulation disorders, hematologic disorders, malignancy, inflammatory bowel disease 2. Medications including chemotherapy, steroids, oral contraceptives	1. Coagulation tests including protein c, protein S, factor V, anticardiolipin antibodies, etc. 2. Movable floating mass attached to the aortic wall on TEE/aortic CTA
**Cardiac sources**			
Takotsubo syndrome	70–90% vs. 10–30% (15–17)	1. Prodromal emotional or physical stressful events, chest pain or discomfort 2. Postmenopausal	1. Dyskinetic ventricle contraction, typically apical ballooning appearance on left ventriculography/echocardiogram 2. Resolution of configuration and function within months of clinical onset
Left atrial appendage aneurysm	52.5 vs. 47.5% (18)	1. Palpitation, dyspnea or arrhythmia	1. Aneurysmal enlargement of the left atrial appendage on echocardiogram/cardiac MRI
Left atrial dissection	55 vs. 45% (19)	1. Recent history of cardiac surgery or intervention 2. Hemodynamically instable symptoms	1. Resembling cardiac tumor or pericardial effusion on TEE/TTE/cardiac MRI
**Venous sources**			
Isolated cortical venous thrombosis	68 vs. 32% (20)	1. Headache, seizure 2. Medical history of coagulation disorders, intracranial hypotension 3. Pregnancy or postpartum 4. Taking oral estrogen containing contraceptives	1. Coagulation tests including protein c, protein S, factor V, anticardiolipin antibodies, etc. 2. Black-blood MRI/MRV/ CTV
May-Thurner syndrome	67 vs. 33% in symptomatic patients (21)	1. Asymmetric lower limb edema, pigmentation 2. Medical history of patent foramen ovale, pulmonary embolism, coagulation disorders 3. Pregnancy or postpartum 4. Taking oral estrogen containing contraceptives	1. Coagulation tests including protein c, protein S, factor V, anticardiolipin antibodies, etc. 2. Compression of iliac vein by the overlying iliac artery and thrombosis on contrast enhanced MRI /contrast enhanced CT of pelvic veins
Pulmonary arteriovenous malformation	60–64% vs. 36–40% (22)	1. Recurrent epistaxis, multifocal cutaneous telangiectasis 2. Medical history of hypoxemia, brain abscess, migraine, decompression illness, hemoptysis and hemothorax 3. Family history of hemorrhagic telangiectasis	1. Positive transcranial doppler bubble test, but no intracardiac shunting in TEE 2. DSA/contrast enhanced chest CT

*CT, computed tomography; CTA, computed tomography angiography; CTV, computed tomography venography; DSA, digital subtraction angiography; MRA, magnetic resonance angiography; MRV, magnetic resonance venography; TEE, transesophageal echocardiogram; TTE, transthoracic echocardiogram*.

## Author Contributions

All authors contributed to the article and approved the submitted version.

## Conflict of Interest

The authors declare that the research was conducted in the absence of any commercial or financial relationships that could be construed as a potential conflict of interest.

## Publisher's Note

All claims expressed in this article are solely those of the authors and do not necessarily represent those of their affiliated organizations, or those of the publisher, the editors and the reviewers. Any product that may be evaluated in this article, or claim that may be made by its manufacturer, is not guaranteed or endorsed by the publisher.
